# 3-Benzoyl-1,5-dimethyl-1*H*-1,5-benzodiazepine-2,4(3*H*,5*H*)-dione

**DOI:** 10.1107/S160053681100866X

**Published:** 2011-03-12

**Authors:** Rachida Dardouri, Youssef Kandri Rodi, Sonia Ladeira, El Mokhtar Essassi, Seik Weng Ng

**Affiliations:** a Laboratoire de Chimie Organique Appliquée, Faculté des Sciences et Techniques Université Sidi Mohamed Ben Abdallah, Fés, Morocco; b Service Commun Rayons-X FR2599, Université Paul Sabatier, Bâtiment 2R1, 118 route de Narbonne, Toulouse, France; c Laboratoire de Chimie Organique Hétérocyclique, Pôle de Compétences Pharmacochimie, Université Mohammed V-Agdal, BP 1014 Avenue Ibn Batout, Rabat, Morocco; d Department of Chemistry, University of Malaya, 50603 Kuala Lumpur, Malaysia

## Abstract

The seven-membered ring of the title compound, C_18_H_16_N_2_O_3_, adopts a boat-shaped conformation (with the C atoms of the fused ring as the stern and the methine C atom as the prow). The substituent at the 3-position occupies an axial position, and the aromatic ring of the substituent is arched over the seven-membered ring in a parasol-like manner, the dihedral angle between the phenyl­ene and phenyl rings being 28.7 (1)°.

## Related literature

For the crystal structure of the 3,3-dimethyl substituted deriv­ative, see: Dardouri *et al.* (2011 ▶).
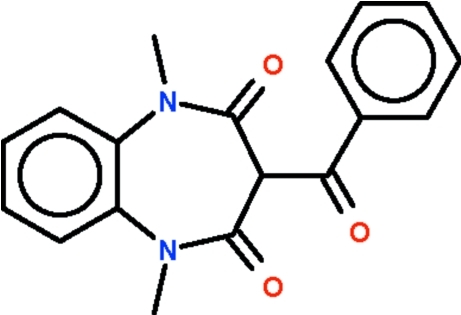


## Experimental

### 

#### Crystal data

C_18_H_16_N_2_O_3_
*M* *_r_* = 308.33
Monoclinic, 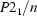

*a* = 7.7827 (1) Å
*b* = 23.7595 (4) Å
*c* = 8.6315 (2) Å
β = 105.614 (1)°
*V* = 1537.18 (5) Å^3^
*Z* = 4
Mo *K*α radiation
μ = 0.09 mm^−1^
*T* = 295 K
0.22 × 0.12 × 0.04 mm

#### Data collection

Bruker X8 APEXII diffractometer
18802 measured reflections
4592 independent reflections
3089 reflections with *I* > 2σ(*I*)
*R*_int_ = 0.038

#### Refinement

*R*[*F* ^2^ > 2σ(*F* ^2^)] = 0.047
*wR*(*F* ^2^) = 0.128
*S* = 1.01
4592 reflections
210 parameters
H-atom parameters constrained
Δρ_max_ = 0.31 e Å^−3^
Δρ_min_ = −0.22 e Å^−3^

### 

Data collection: *APEX2* (Bruker, 2008 ▶); cell refinement: *SAINT* (Bruker, 2008 ▶); data reduction: *SAINT*; program(s) used to solve structure: *SHELXS97* (Sheldrick, 2008 ▶); program(s) used to refine structure: *SHELXL97* (Sheldrick, 2008 ▶); molecular graphics: *X-SEED* (Barbour, 2001 ▶); software used to prepare material for publication: *publCIF* (Westrip, 2010 ▶).

## Supplementary Material

Crystal structure: contains datablocks global, I. DOI: 10.1107/S160053681100866X/bt5487sup1.cif

Structure factors: contains datablocks I. DOI: 10.1107/S160053681100866X/bt5487Isup2.hkl

Additional supplementary materials:  crystallographic information; 3D view; checkCIF report

## Figures and Tables

**Fig. 1.**
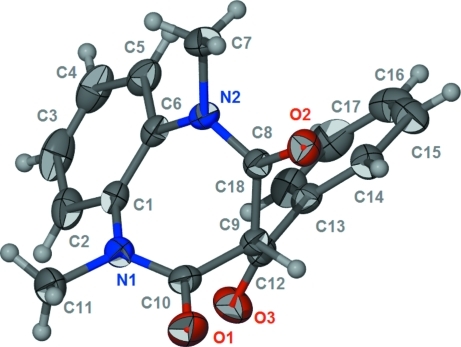
Displacement ellipsoid plot (Barbour, 2001) of C18H16N2O3 at the 70% probability level; hydrogen atoms are drawn as arbitrary radius.

**Fig. 2.**
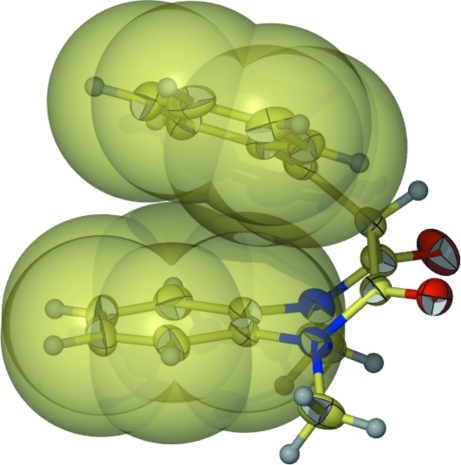
Van der Waals surfaces of the carbon atoms of the aromatic rings.

## supplementary crystallographic information

### Comment

The methylene part of 1,5-dimethyl-1,5-benzodiazepine-2,4-dione is relatively acidic, and one proton can be abstracted by using potassium *t*-butoxide; the resulting carbanion can undergo a nucleophlilic substitution with an alkyl halide to form 3-substituted derivatives. A previous study reported the crystal structure of the 3,3-dimethyl-substituted derivative, which was synthesized by a slight variation of the synthetic route (Dardouri *et al.*, 2011). The title compound was obtained by using benzoyl chloride as reactant. The seven-membered ring of C_18_H_16_N_2_O_3_ adopts a boat-shaped conformation (with the C atoms of the fused-ring as the stern and the methine C atom as the prow) (Scheme I, Fig. 1). The substituent at the 3-position occupies an axial position. The unfavorable the 3-position forces the phenyl ring to arch over the phenylene ring of the fused-ring in a parasol-like manner [the dihedral angle between the two rings is 28.7 (1) °]. The distance between the two centroids is 4.225 Å (Fig. 2). Severe strain is also evident from the non-linearity of the benzoyl C_6_H_5_C(O)– portion of the molecule.

### Experimental

To a solution of potassium *t*-butoxide (0.42 g, 3.6 mmol) in DMF (15 ml) was added 1,5-dimethyl-1,5-benzodiazepine-2,4-dione (0.50 g, 2.4 mmol) and benzoyl chloride (0.33 ml, 2.88 mmol). Stirring was continued for 24 h. The reaction was monitored by thin layer chromatography. The mixture was filtered; slow evaporation of the solvent gave colorless crystals.

### Refinement

H-atoms were placed in calculated positions (C—H 0.93–0.97 Å) and were included in the refinement in the riding model approximation, with *U*(H) set to 1.2–1.5*U*_eq_(C).

Omitted from the refinement was the (6 0 6) reflection owing to bad agreement between observed and calculated structure factor.

### Crystal data

**Table:**

C_18_H_16_N_2_O_3_	*F*(000) = 648
*M**_r_* = 308.33	*D*_x_ = 1.332 Mg m^−^^3^
Monoclinic, *P*2_1_/*n*	Mo *K*α radiation, λ = 0.71073 Å
Hall symbol: -P 2yn	Cell parameters from 4206 reflections
*a* = 7.7827 (1) Å	θ = 2.6–28.3°
*b* = 23.7595 (4) Å	µ = 0.09 mm^−^^1^
*c* = 8.6315 (2) Å	*T* = 295 K
β = 105.614 (1)°	Prism, colorless
*V* = 1537.18 (5) Å^3^	0.22 × 0.12 × 0.04 mm
*Z* = 4	

### Data collection

**Table:**

Bruker X8 APEXII diffractometer	3089 reflections with *I* > 2σ(*I*)
Radiation source: fine-focus sealed tube	*R*_int_ = 0.038
graphite	θ_max_ = 30.5°, θ_min_ = 2.6°
φ and ω scans	*h* = −10→9
18802 measured reflections	*k* = −33→30
4592 independent reflections	*l* = −12→11

### Refinement

**Table:**

Refinement on *F*^2^	Primary atom site location: structure-invariant direct methods
Least-squares matrix: full	Secondary atom site location: difference Fourier map
*R*[*F*^2^ > 2σ(*F*^2^)] = 0.047	Hydrogen site location: inferred from neighbouring sites
*wR*(*F*^2^) = 0.128	H-atom parameters constrained
*S* = 1.01	*w* = 1/[σ^2^(*F*_o_^2^) + (0.0632*P*)^2^ + 0.2366*P*] where *P* = (*F*_o_^2^ + 2*F*_c_^2^)/3
4592 reflections	(Δ/σ)_max_ = 0.001
210 parameters	Δρ_max_ = 0.31 e Å^−^^3^
0 restraints	Δρ_min_ = −0.22 e Å^−^^3^

### Special details

**Table:**

Geometry. All e.s.d.'s (except the e.s.d. in the dihedral angle between two l.s. planes) are estimated using the full covariance matrix. The cell e.s.d.'s are taken into account individually in the estimation of e.s.d.'s in distances, angles and torsion angles; correlations between e.s.d.'s in cell parameters are only used when they are defined by crystal symmetry. An approximate (isotropic) treatment of cell e.s.d.'s is used for estimating e.s.d.'s involving l.s. planes.

### Fractional atomic coordinates and isotropic or equivalent isotropic displacement parameters (Å^2^)

**Table:**

	*x*	*y*	*z*	*U*_iso_*/*U*_eq_	
O1	1.24318 (14)	0.44276 (5)	0.90455 (13)	0.0423 (3)	
O2	0.83292 (13)	0.51285 (4)	0.61289 (12)	0.0311 (2)	
O3	1.09190 (15)	0.33492 (5)	0.61730 (13)	0.0385 (3)	
N1	1.01821 (15)	0.38528 (5)	0.92487 (13)	0.0268 (3)	
N2	0.71073 (14)	0.43944 (5)	0.71264 (13)	0.0245 (2)	
C1	0.85958 (18)	0.35496 (6)	0.85124 (15)	0.0250 (3)	
C2	0.8500 (2)	0.29767 (6)	0.88450 (18)	0.0353 (3)	
H2	0.9505	0.2793	0.9467	0.042*	
C3	0.6934 (3)	0.26792 (7)	0.82626 (19)	0.0428 (4)	
H3	0.6882	0.2300	0.8512	0.051*	
C4	0.5446 (2)	0.29455 (7)	0.73107 (19)	0.0415 (4)	
H4	0.4385	0.2747	0.6934	0.050*	
C5	0.5533 (2)	0.35047 (7)	0.69189 (17)	0.0333 (3)	
H5	0.4536	0.3678	0.6250	0.040*	
C6	0.70996 (17)	0.38152 (5)	0.75123 (15)	0.0237 (3)	
C7	0.5560 (2)	0.47429 (7)	0.7181 (2)	0.0381 (4)	
H7A	0.5945	0.5122	0.7462	0.057*	
H7B	0.5021	0.4594	0.7972	0.057*	
H7C	0.4706	0.4740	0.6145	0.057*	
C8	0.84081 (17)	0.46368 (5)	0.65773 (15)	0.0225 (3)	
C9	1.00564 (16)	0.42783 (5)	0.66442 (15)	0.0231 (3)	
H9	1.0844	0.4514	0.6204	0.028*	
C10	1.10278 (18)	0.41905 (6)	0.84130 (16)	0.0272 (3)	
C11	1.1054 (2)	0.37505 (8)	1.09563 (17)	0.0407 (4)	
H11A	1.0189	0.3616	1.1478	0.061*	
H11B	1.1567	0.4095	1.1455	0.061*	
H11C	1.1976	0.3474	1.1053	0.061*	
C12	0.98410 (18)	0.37241 (5)	0.56927 (16)	0.0246 (3)	
C13	0.84063 (18)	0.36688 (5)	0.41676 (16)	0.0246 (3)	
C14	0.77679 (19)	0.41305 (6)	0.31835 (16)	0.0290 (3)	
H14	0.8199	0.4489	0.3503	0.035*	
C15	0.6493 (2)	0.40561 (7)	0.17305 (18)	0.0370 (4)	
H15	0.6087	0.4363	0.1065	0.044*	
C16	0.5825 (2)	0.35255 (8)	0.12720 (19)	0.0404 (4)	
H16	0.4957	0.3477	0.0303	0.048*	
C17	0.6441 (2)	0.30642 (7)	0.2249 (2)	0.0418 (4)	
H17	0.5981	0.2708	0.1938	0.050*	
C18	0.7740 (2)	0.31344 (6)	0.36851 (18)	0.0335 (3)	
H18	0.8169	0.2824	0.4330	0.040*	

### Atomic displacement parameters (Å^2^)

**Table:**

	*U*^11^	*U*^22^	*U*^33^	*U*^12^	*U*^13^	*U*^23^
O1	0.0235 (5)	0.0623 (8)	0.0383 (6)	−0.0123 (5)	0.0034 (5)	0.0014 (5)
O2	0.0369 (6)	0.0235 (5)	0.0353 (5)	0.0024 (4)	0.0137 (4)	0.0040 (4)
O3	0.0413 (6)	0.0364 (6)	0.0379 (6)	0.0173 (5)	0.0107 (5)	0.0034 (4)
N1	0.0231 (6)	0.0338 (6)	0.0230 (5)	−0.0002 (5)	0.0052 (4)	0.0028 (4)
N2	0.0203 (5)	0.0269 (6)	0.0278 (6)	0.0017 (4)	0.0093 (4)	0.0025 (4)
C1	0.0277 (7)	0.0265 (6)	0.0237 (6)	−0.0017 (5)	0.0118 (5)	0.0008 (5)
C2	0.0484 (9)	0.0274 (7)	0.0357 (8)	0.0038 (6)	0.0210 (7)	0.0055 (6)
C3	0.0678 (12)	0.0260 (7)	0.0434 (9)	−0.0143 (7)	0.0303 (9)	−0.0051 (6)
C4	0.0504 (10)	0.0427 (9)	0.0365 (8)	−0.0250 (8)	0.0206 (8)	−0.0122 (7)
C5	0.0293 (7)	0.0438 (9)	0.0276 (7)	−0.0116 (6)	0.0094 (6)	−0.0047 (6)
C6	0.0246 (7)	0.0259 (6)	0.0229 (6)	−0.0038 (5)	0.0106 (5)	−0.0007 (5)
C7	0.0279 (8)	0.0460 (9)	0.0451 (9)	0.0127 (6)	0.0179 (7)	0.0107 (7)
C8	0.0226 (6)	0.0246 (6)	0.0200 (6)	−0.0014 (5)	0.0052 (5)	−0.0009 (5)
C9	0.0201 (6)	0.0250 (6)	0.0256 (6)	−0.0009 (5)	0.0087 (5)	0.0020 (5)
C10	0.0209 (6)	0.0335 (7)	0.0275 (6)	0.0002 (5)	0.0070 (5)	0.0003 (5)
C11	0.0335 (8)	0.0628 (11)	0.0243 (7)	0.0016 (7)	0.0053 (6)	0.0088 (7)
C12	0.0252 (6)	0.0251 (6)	0.0267 (6)	0.0020 (5)	0.0122 (5)	0.0030 (5)
C13	0.0260 (7)	0.0240 (6)	0.0273 (6)	0.0021 (5)	0.0134 (5)	−0.0016 (5)
C14	0.0341 (8)	0.0254 (7)	0.0282 (7)	0.0041 (6)	0.0093 (6)	−0.0007 (5)
C15	0.0393 (9)	0.0431 (9)	0.0274 (7)	0.0141 (7)	0.0070 (6)	−0.0026 (6)
C16	0.0312 (8)	0.0568 (10)	0.0326 (8)	0.0029 (7)	0.0077 (6)	−0.0165 (7)
C17	0.0424 (9)	0.0387 (9)	0.0473 (9)	−0.0129 (7)	0.0173 (8)	−0.0181 (7)
C18	0.0417 (8)	0.0252 (7)	0.0374 (8)	−0.0012 (6)	0.0171 (7)	−0.0035 (6)

### Geometric parameters (Å, °)

**Table:**

O1—C10	1.2199 (17)	C7—H7C	0.9600
O2—C8	1.2271 (16)	C8—C9	1.5280 (17)
O3—C12	1.2173 (16)	C9—C10	1.5247 (18)
N1—C10	1.3613 (17)	C9—C12	1.5369 (18)
N1—C1	1.4229 (18)	C9—H9	0.9800
N1—C11	1.4675 (18)	C11—H11A	0.9600
N2—C8	1.3562 (16)	C11—H11B	0.9600
N2—C6	1.4163 (17)	C11—H11C	0.9600
N2—C7	1.4725 (17)	C12—C13	1.4851 (19)
C1—C2	1.3974 (19)	C13—C18	1.3922 (19)
C1—C6	1.3993 (19)	C13—C14	1.3945 (19)
C2—C3	1.381 (2)	C14—C15	1.385 (2)
C2—H2	0.9300	C14—H14	0.9300
C3—C4	1.380 (3)	C15—C16	1.380 (2)
C3—H3	0.9300	C15—H15	0.9300
C4—C5	1.377 (2)	C16—C17	1.387 (2)
C4—H4	0.9300	C16—H16	0.9300
C5—C6	1.3986 (19)	C17—C18	1.383 (2)
C5—H5	0.9300	C17—H17	0.9300
C7—H7A	0.9600	C18—H18	0.9300
C7—H7B	0.9600		
			
C10—N1—C1	123.15 (11)	C8—C9—C12	119.19 (11)
C10—N1—C11	118.11 (12)	C10—C9—H9	105.8
C1—N1—C11	118.41 (11)	C8—C9—H9	105.8
C8—N2—C6	123.07 (11)	C12—C9—H9	105.8
C8—N2—C7	117.79 (11)	O1—C10—N1	122.53 (13)
C6—N2—C7	118.91 (11)	O1—C10—C9	121.87 (12)
C2—C1—C6	118.98 (13)	N1—C10—C9	115.53 (11)
C2—C1—N1	119.29 (13)	N1—C11—H11A	109.5
C6—C1—N1	121.68 (12)	N1—C11—H11B	109.5
C3—C2—C1	120.95 (15)	H11A—C11—H11B	109.5
C3—C2—H2	119.5	N1—C11—H11C	109.5
C1—C2—H2	119.5	H11A—C11—H11C	109.5
C4—C3—C2	119.93 (14)	H11B—C11—H11C	109.5
C4—C3—H3	120.0	O3—C12—C13	121.46 (12)
C2—C3—H3	120.0	O3—C12—C9	118.61 (12)
C3—C4—C5	119.98 (14)	C13—C12—C9	119.82 (11)
C3—C4—H4	120.0	C18—C13—C14	119.49 (13)
C5—C4—H4	120.0	C18—C13—C12	118.46 (12)
C4—C5—C6	120.98 (15)	C14—C13—C12	122.00 (12)
C4—C5—H5	119.5	C15—C14—C13	120.13 (13)
C6—C5—H5	119.5	C15—C14—H14	119.9
C5—C6—C1	119.11 (13)	C13—C14—H14	119.9
C5—C6—N2	119.00 (12)	C16—C15—C14	119.93 (15)
C1—C6—N2	121.85 (11)	C16—C15—H15	120.0
N2—C7—H7A	109.5	C14—C15—H15	120.0
N2—C7—H7B	109.5	C15—C16—C17	120.36 (14)
H7A—C7—H7B	109.5	C15—C16—H16	119.8
N2—C7—H7C	109.5	C17—C16—H16	119.8
H7A—C7—H7C	109.5	C18—C17—C16	119.94 (14)
H7B—C7—H7C	109.5	C18—C17—H17	120.0
O2—C8—N2	122.40 (12)	C16—C17—H17	120.0
O2—C8—C9	120.86 (11)	C17—C18—C13	120.12 (14)
N2—C8—C9	116.62 (11)	C17—C18—H18	119.9
C10—C9—C8	107.40 (10)	C13—C18—H18	119.9
C10—C9—C12	111.80 (11)		
			
C10—N1—C1—C2	130.65 (14)	N2—C8—C9—C12	60.87 (15)
C11—N1—C1—C2	−42.59 (18)	C1—N1—C10—O1	−176.50 (13)
C10—N1—C1—C6	−51.95 (18)	C11—N1—C10—O1	−3.2 (2)
C11—N1—C1—C6	134.80 (14)	C1—N1—C10—C9	6.51 (18)
C6—C1—C2—C3	−3.0 (2)	C11—N1—C10—C9	179.78 (12)
N1—C1—C2—C3	174.49 (13)	C8—C9—C10—O1	−107.48 (15)
C1—C2—C3—C4	1.3 (2)	C12—C9—C10—O1	120.01 (14)
C2—C3—C4—C5	1.2 (2)	C8—C9—C10—N1	69.54 (14)
C3—C4—C5—C6	−2.0 (2)	C12—C9—C10—N1	−62.97 (15)
C4—C5—C6—C1	0.3 (2)	C10—C9—C12—O3	−26.52 (16)
C4—C5—C6—N2	−177.66 (13)	C8—C9—C12—O3	−152.84 (12)
C2—C1—C6—C5	2.15 (19)	C10—C9—C12—C13	157.22 (11)
N1—C1—C6—C5	−175.25 (12)	C8—C9—C12—C13	30.89 (16)
C2—C1—C6—N2	−179.97 (12)	O3—C12—C13—C18	30.37 (19)
N1—C1—C6—N2	2.63 (19)	C9—C12—C13—C18	−153.48 (12)
C8—N2—C6—C5	−131.99 (13)	O3—C12—C13—C14	−146.95 (14)
C7—N2—C6—C5	42.36 (17)	C9—C12—C13—C14	29.20 (18)
C8—N2—C6—C1	50.13 (18)	C18—C13—C14—C15	−0.6 (2)
C7—N2—C6—C1	−135.52 (13)	C12—C13—C14—C15	176.69 (13)
C6—N2—C8—O2	174.18 (12)	C13—C14—C15—C16	1.4 (2)
C7—N2—C8—O2	−0.23 (19)	C14—C15—C16—C17	−0.9 (2)
C6—N2—C8—C9	−9.73 (17)	C15—C16—C17—C18	−0.4 (2)
C7—N2—C8—C9	175.85 (12)	C16—C17—C18—C13	1.2 (2)
O2—C8—C9—C10	108.64 (13)	C14—C13—C18—C17	−0.7 (2)
N2—C8—C9—C10	−67.51 (14)	C12—C13—C18—C17	−178.09 (13)
O2—C8—C9—C12	−122.98 (13)		
